# Fungal Laccases: The Forefront of Enzymes for Sustainability

**DOI:** 10.3390/jof7121048

**Published:** 2021-12-07

**Authors:** Martina Loi, Olga Glazunova, Tatyana Fedorova, Antonio F. Logrieco, Giuseppina Mulè

**Affiliations:** 1 Institute of Sciences of Food Production, National Research Council, Via Amendola 122/O, 70126 Bari, Italy; martina.loi@ispa.cnr.it (M.L.); antonio.logrieco@ispa.cnr.it (A.F.L.); 2 A.N. Bach Institute of Biochemistry, Research Center of Biotechnology of the Russian Academy of Sciences, 119071 Moscow, Russia; olga.a.glas@gmail.com (O.G.); fedorova_tv@mail.ru (T.F.)

**Keywords:** enzymes, sustainability, fungal laccase, solid state fermentation, agro-wastes, immobilization, catalysis

## Abstract

Enzymatic catalysis is one of the main pillars of sustainability for industrial production. Enzyme application allows minimization of the use of toxic solvents and to valorize the agro-industrial residues through reuse. In addition, they are safe and energy efficient. Nonetheless, their use in biotechnological processes is still hindered by the cost, stability, and low rate of recycling and reuse. Among the many industrial enzymes, fungal laccases (LCs) are perfect candidates to serve as a biotechnological tool as they are outstanding, versatile catalytic oxidants, only requiring molecular oxygen to function. LCs are able to degrade phenolic components of lignin, allowing them to efficiently reuse the lignocellulosic biomass for the production of enzymes, bioactive compounds, or clean energy, while minimizing the use of chemicals. Therefore, this review aims to give an overview of fungal LC, a promising green and sustainable enzyme, its mechanism of action, advantages, disadvantages, and solutions for its use as a tool to reduce the environmental and economic impact of industrial processes with a particular insight on the reuse of agro-wastes.

## 1. Introduction

Enzyme biotechnology is a green and efficient process which has been applied to various industrial operations as an alternative to chemical catalysis with advantages in terms of sustainability and efficiency [1]. Indeed, enzymatic catalysis enables the tackling of most issues that are related to environmental sustainability, especially regarding the use of hazardous chemicals, and represents an important tool that may be applied at an industrial level.

The use of enzymes may significantly reduce the environmental impact of industrial operations. Nonetheless, enzyme production and formulation are energy- and labor-intensive processes, which may incur in environmental impacts as well. In addition, techno-economic analyses of enzyme production revealed that the technological readiness and process economics for enzyme scale-up and use in industrial processes are not always encouraging [1]. Sustainability refers to all actions devoted to satisfying the needs of present generations without jeopardizing the ability of future generations to meet their own needs. Hence, sustainability is a complex and holistic concept which brings together the economic, social, and environmental fields [2]. Sustainability in industry encompasses the reduction of pollution that is caused by chemicals; the hazards deriving from their use; the utilization of abundant, carbon-neutral biomasses or wastes as raw materials (i.e., circularity); the use of renewable energy sources; and the use of efficient conversion technologies [3].

Today the synthesis, conversion, removal, or remediation of chemicals is often performed by means of enzyme biotechnology in different sectors, such as pharmaceutical, food and beverage, feed, biofuel, textile, paper and pulp, and leather industries. Unsurprisingly, the global enzyme market has been increasing in recent years. It was valued USD 9.9 billion in 2019 [4] and it is expected to continue growing at a compound annual growth rate of 6.8–7.1%, drawn in by food, beverages, laundry, and detergents industries [4,5].

Approximately 60% of industrial enzymes are produced from fungi, 24% from bacteria, 4% from yeasts, and the remaining 10% from plants and animals [6]. Hydrolases account for 75% of the entire enzyme production worldwide [6].

Lignin-modifying enzymes from white-rot fungi are the forefront of enzyme technology for sustainability because they allow the degradation of the phenolic components of lignin and release the cellulose that is used in microbial fermentations to produce energy or valuable bioactive ingredients [7].

Owing to the enzymatic treatment, the shift from energy intensive processes, such as steam or chemical-based ones, to mild and environmentally friendly processes is feasible [8], while valorizing the plant material and applying circularity in food systems. In this regard, plant material deriving from food wastes still represents a poorly exploited resource which may enter new production paths. Indeed, the Food and Agriculture Organization estimated that in 2019 17% of total food that was available to consumers was discarded at household, retailer, and restaurant levels [9,10].

Biobased methods for the breakdown of lignocellulosic biomasses are essential to allow the complete exploitation of cellulose and its further valorization and are gaining massive popularity nowadays.

Laccase (LC, benzenediol: oxygen oxidoreductases, EC 1.10.3.2) is a multicopper oxidase which has a long history of application in de-lignification, lignocellulosic fiber modification, chemical synthesis, and many other industrial applications [11]. LCs have also shown great potential in the bioremediation of toxic pollutants such as dyes [12,13], polycyclic aromatic hydrocarbons [14], endocrine disruptors [15], and mycotoxins [16]. It is worth mentioning that this is of particular interest for the reuse of material which can be potentially contaminated and unsafe to use. Its versatility and environmentally friendly catalysis opens up countless possibilities for the valorization of such material in the era of sustainability.

Therefore, this review aims to give an overview of fungal LC, a promising green and sustainable enzyme, its mechanism of action, advantages, disadvantages, and solutions for its use as a tool to reduce the environmental and economic impact of industrial processes with a particular insight on the reuse and valorization of agro-wastes.

## 2. Laccase: A Case Study

### 2.1. Laccase: Overview, Sources

Laccase (p-diphenol: dioxygen oxidoreductase EC 1.10.3.2) belongs to the group of multicopper oxidases (MCOs) that catalyze one-electron oxidation of a wide range of compounds with concomitant reduction of molecular oxygen to water. Electron donor substrates for laccases can be substituted monophenols, polyphenols, aromatic amines, aromatic thiols, as well as some other aromatic compounds and certain inorganic metal ions [17].

Laccase was first isolated over 100 years ago from the Chinese lacquer tree (*Rhus vernicifera*) [17]. A decade later, the first fungal laccase was described [18]. For a long time, laccase was considered as an enzyme that was present only in fungi or higher plants, but later laccases were also found in bacteria, insects, and algae [19]. To date, according to the BRENDA database (http://www.brenda-enzymes.org/ accessed on 24 June 2021), about 280 laccases have been isolated and characterized. However, the modern classification of laccases that is based on phylogenetic analysis considers only fungal (basidiomycetous and ascomycetous) laccases as “true” laccases (laccases *sensu stricto*) [20].

The issue of the biological (or physiological) role of laccases is still controversial. Laccases can perform a variety of functions in different organisms including both the processes of synthesis of new compounds from simple monomeric substrates and the destruction of polymeric substrates. However, it is believed that the main biological function of laccases is lignification in higher plants and delignification in fungi. Other functions that have been proposed for laccases are participating in morphogenesis, pathogenesis, fruiting body formation, sporulation, spore protection, humification, metal ion homeostasis, and others [21,22,23].

A typical fungal genome contains several nonallelic laccase genes which form a multigene family [24]. However, the biological role of various laccase izoenzymes (i.e., the products of different genes) is still unclear. Some izoenzymes could be secreted by a fungus under specific conditions while others may be obtained only by recombinant expression [25]. In addtition, post-translational modification of laccase proteins (i.e., glycosylation) leads to a wide spectrum of laccase isoforms which can be produced by fungi.

The active site of the laccase contains four copper ions which are classified according to their spectral characteristics as copper ions of the T1, T2, and T3 types. The T1 copper ions are characterized by strong absorption at 605–610 nm. In the oxidized state, it gives laccases a characteristic blue color. The T3 copper ions are characterized by a shoulder at 330 nm, while the T2 copper ions have no signal in the absorption spectra. In EPR spectra, T1 and T2 copper ions are characterized by hyperfine splitting, while a pair of T3 copper ions has no signal due to strong anti-ferromagnetic coupling [26]. The oxidation of the phenolic (and other electron donor) substrates takes place at the mononuclear T1 center which contains one T1 copper ion (Cu1). This copper ion serves as the primary electron acceptor. Molecular oxygen is reduced to water at the trinuclear T2/T3 centers which is formed by one T2 copper ion (Cu2) and two T3 copper ions (Cu3).

An important characteristic of laccases is the redox potential of the T1 copper center of the enzyme. The value of the redox potential determines whether a substrate can be oxidized by laccase. In some cases (mostly for the substrates with a redox potential that is higher than 700 mV), the efficiency of substrate oxidation by laccase directly depends on the difference between the redox potentials of the substrate and the T1 center of the enzyme [27,28]. However, the efficiency of catalysis is influenced not only by the redox potential difference, but also by the structure of the substrate-binding pocket of the laccase and the structure of the substrate [28,29,30].

Depending on the value of their redox potential, laccases are usually divided into enzymes with low, medium, and high redox potential [31]. The redox potential of fungal laccases is, as a rule, higher than that of enzymes of plant and bacterial origin [32]. The group of enzymes with a low redox potential (up to 460 mV vs. NHE) includes mainly plant and bacterial laccases, the group with medium redox potential (460–710 mV vs. NHE) includes laccases of *Ascomycete* and *Basidiomycete* fungi, whereas high redox potential (more than 710 mV vs. NHE) is characteristic mainly of laccases of wood-degrading white-rot fungi [33,34].

The redox potential of the T2/T3 center affects catalysis of laccases to a lesser extent [35]. The redox potential value of the T2 center was measured for laccases of *Trametes hirsuta* (400 mV) [36] and *R. vernicifera* (365 mV) [37]. The redox potential value of the T3 center is 756 mV for the laccase of *T. hirsuta* [38] and 434 mV for the laccase of *R. vernicifera* [37].

### 2.2. Physicochemical and Catalytic Properties, Enzyme Stability

Nowadays, the most studied groups are the fungal and bacterial laccases. Most laccases are glycoproteins with a molecular weight of 50–140 kDa [31,32,33,34,35,36,37,38,39]. Usually, laccases exist in a monomeric form but there are also laccases that form homo- and hetero-dimers. The isoelectric points of laccases are in a wide range of pH from 2.6 to 7.0. Fungal laccases are characterized by isoelectric points near 4.0, while plant laccases, as a rule, have neutral pI values [39,40].

The carbohydrate part of a laccase molecule can comprise of up to 50% of the total mass of the protein; plant laccases are characterized by a higher carbohydrate content (usually from 20 to 45%), while fungal laccases have a slightly lower carbohydrate content (usually from 10 to 25%) [41]. N-glycosylation is almost always found in fungal laccases. Branched mannose chains are bonded to asparagine residues via two N-acetylglucosamine residues at 3–10 sites [35]. Presumably, the role of carbohydrates is to stabilize copper centers, participate in the process of protein secretion, protect the molecule from proteolysis, and increase the thermal stability [42,43]. Moreover, glycosylation can affect the activity of laccases [44,45,46,47]. Differences in glycosylation can also be responsible for the presence of a large number of laccase isoforms differing in molecular weight and pI values [48,49,50].

For fungal laccases, it is typical to have an acidic pH optima. The optimum for 2,2′-azinobis(3-ethylbenzthiazoline-6-sulfonic acid) (ABTS) oxidation is generally below pH 4.0, while the oxidation optima for phenolic compounds such as 2,6-dimethoxyphenol, guaiacol, and syringaldazine are in the pH range from 4.0 to 7.0 [40].

Typically, the temperature optimum for laccase activity is in the range from 50 to 70 °C. Some laccases have a temperature optimum that is below 35 °C [40]. The thermal stability of laccases that are isolated from different sources varies greatly, even in laccases that are from different isolates of the same species [42]. The thermal stability of laccases depends on many physicochemical factors—the packaging of the protein globule, the hydrophobicity, the number of intramolecular hydrogen bonds and salt bridges, the distribution of charged amino acid residues on the protein surface, or an increased content of certain amino acid residues [42].

Laccases are capable of catalyzing the oxidation of a very wide range of substrates including various xenobiotic organic compounds such as aromatic amines, polycyclic aromatic hydrocarbons, synthetic dyes, antibiotics, etc. [19,25]. However, the typical substrates for laccases are various substituted phenols. The efficiency of catalysis of various compounds is also influenced by the number and nature of substituents in the phenolic ring [51]. Due to a higher redox potential, laccases of basidiomycetes have a broader substrate specificity in comparison with laccases of ascomycetes, plants, and bacteria [52].

**Figure 1:**
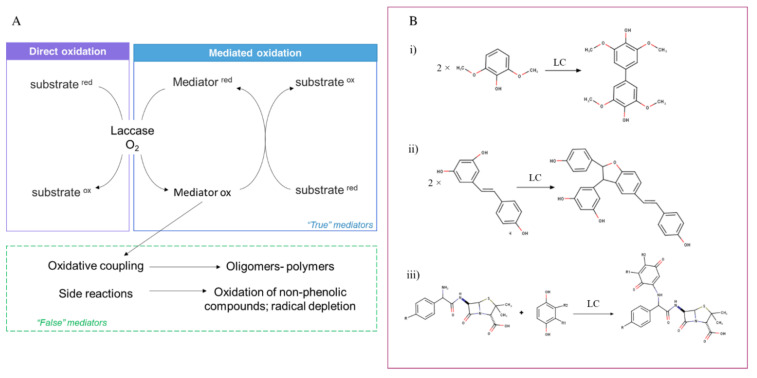
Oxidation of substrates by laccase. Panel *(***A***)* shows the direct and mediated oxidation mechanisms. The route of “false” mediators is also depicted. Panel *(***B***)* shows three examples of coupling reactions. In particular homomolecular coupling of 2,6-dimethoxyphenol (i) and resveratrol (ii), and the heteromolecular coupling of aminopenicillin and cathecol (iii).

Additionally, the substrate specificity of laccases can be extended by using mediators —low molecular weight compounds that act as electron shuttles. It is suggested that mediators can promote catalysis through two different mechanisms. First, in reactions with compounds whose direct oxidation by laccase is sterically hindered due to their big size or/and complex structure, mediators can enable substrate oxidation by acting as electron transfer agents. Second, in reactions with compounds whose direct oxidation by laccase is impossible due to their high redox potential, mediators can enable substrate oxidation by promoting alternative (non-enzymatic) oxidative pathways [53,54,55,56]. From the chemical perspective, laccase mediator should be a small-sized compound which could be transformed by laccase into a stable but still reactive oxidized form and then reduced to the initial form by the final substrate; hence recycling during the reaction. It should be noted that most of the mediators that are described in the literature are not true redox mediators because they undergo secondary chemical reactions (i.e., are consumed during the reaction cycle). Among the secondary reactions, the coupling reactions are of great relevance, especially in organic synthesis as they allow the production of novel compounds. Homomolecular coupling in the reaction consists of two oxidized substrates to form a dimer or an oligomer. Heteromolecular coupling involves an oxidized LC substrate and a non-laccase) to create new hybrid molecules [55]. Figure 1A shows the scheme of laccase oxidation of phenolic derivatives with and without the participation of a mediator. In addition, three examples of reaction couplings are reported (Figure 1B).

An example of “false” mediators is represented by natural phenolic compounds which can undergo polymerization reactions. The synthetic mediators HBT and VA also undergo chemical degradation after a few cycles. True mediators include a very limited number of compounds, including inorganic ion complexes (potassium octocyanomolybdate and octocyanotungstate, FeII complexes) and organic compounds (ABTS, TEMPO) [55].

### 2.3. Laccase Structure and Mechanism of Catalysis

**Figure 2:**
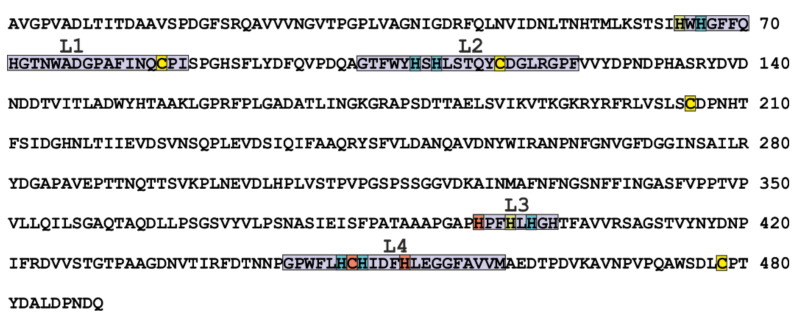
Amino acid sequence of *T. hirsuta* LacA laccase (gb | KP027478.1). The L1-L4 regions are shown in purple, the copper ion Cu1 ligands are shown in orange, Cu2—in green, and Cu3—in blue. Cysteine residues that are involved in the formation of disulfide bonds are shown in yellow.

Usually, amino acid sequences of laccases, *sensu stricto*, contain 520–550 residues including signal peptide [32]; it is worth noting that the sequence identity of laccases of organisms that belong to different classes is 20–30%. Multiple alignment of amino acid sequences of more than 100 fungal and plant laccases allowed the identification of four conserved regions (L1–L4) distinguishing laccases from other proteins from the MCO family [57]. Figure 2 shows the sequence of the laccase LacA from *T. hirsuta* with the regions L1–L4 highlighted in purple. These regions contain 12 amino acid residues coordinating the copper ions in the active site of the enzyme.

**Figure 3:**
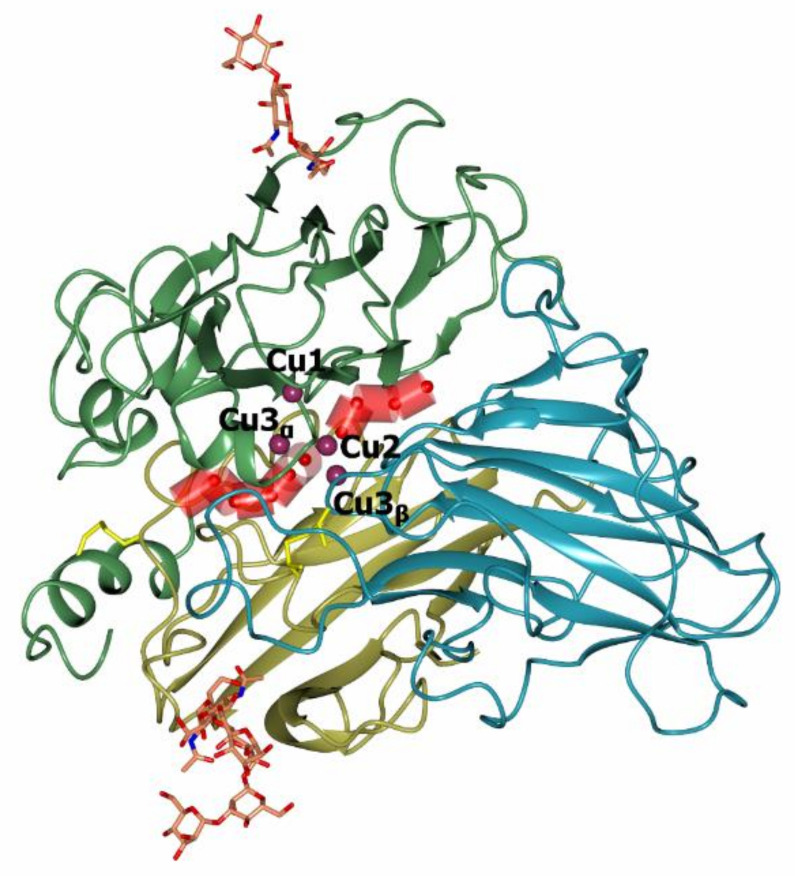
The overall structure of *Coriolopsis caperata* laccase (PDB 3JHV, [60]). The first domain is shown in gold, the second in green, and the third in blue color. Copper atoms are shown with purple spheres. Sugars are shown with stick models, atoms are colored by type (C—orange, O—red, N—blue). Disulfide bridges are shown in yellow.

The first three-dimensional structure was determined in 1998 for a laccase from the fungus *Coprinus cinereus* [58]. At present, the PDB database (www.rcsb.org, accessed on 24 June 2021) contains more than 180 structures of laccases of bacteria (17 species) and fungi (25 species). The 3D structures of fungal laccases are very similar to those of other MCOs, such as ascorbate oxidase, bilirubin oxidase, and ceruloplasmin. Three cupredoxin-like domains are sequentially connected to each other: domain I contains ~1–130 amino acid residues, domain II—~130 and 310 residues, and domain III—~310–500 residues (Figure 3). Each domain has the Greek key β barrel topology which is typical for all proteins of the MCO family [59,60]. As a rule, the structure is stabilized at least by two disulphide bridges connecting the first domain with the other two domains. The T1 center is located in domain III, while the T2/T3 center (TNC) is located between domains I and II. The distance between the T1 and T2/T3 centers is approximately 13 Å, but they are connected via a conservative His-Cys-His motif that is involved in the electron transfer from the T1 center to the TNC [23]. Bacterial laccases can have a three-domain structure, similar to the fungal laccases. However, in addition to three-domain laccases, there are also two-domain bacterial laccases which form trimers [59].

**Figure 4:**
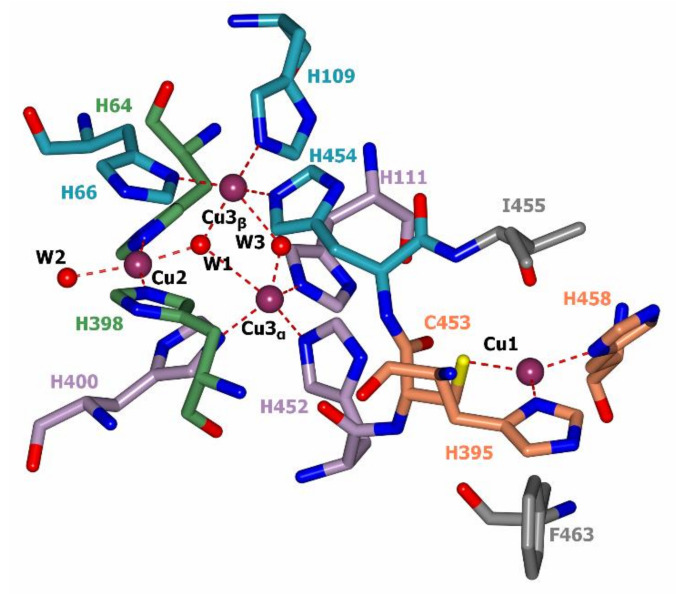
Active center of *T. hirsuta* laccase (PDB: 3FPX, [61]). Copper ions are shown in purple, oxygen atoms in red, and nitrogen atoms in blue. The carbon atoms of histidine residues coordinating copper ions Cu1, Cu2, Cu3_α_, and Cu3_β_ are shown in coral, green, purple, and blue colors, respectively. Carbon atoms of non-coordinating amino acid residues from the nearest surrounding of the copper ion Cu1 are shown in gray. T2 and T3 water channels are shown with red cylinders and water molecules inside them.

In all laccases there is an identical structure of the T1 center where the oxidation of the electron donor substrate occurs. The copper ion in the T1 center is coordinated by the side chains of two histidine residues and a cysteine residue (Figure 4). Moreover, in the vicinity of the Cu1 copper ion (~5 Å), the side chains of another two amino acid residues are located (highlighted in gray in Figure 4). One of them, isoleucine, is strictly conservative among laccases. In fungal laccases, the second position is usually occupied by phenylalanine or leucine residues, whereas in bacterial laccases a methionine residue is located there [61]. There is a correlation between the redox potential of laccases and the nature of the non-conservative amino acid residue in the nearest surrounding of the copper ion in the T1 active center. High redox potential laccases have a Phe residue in this position, occasionally Leu, middle redox potential laccases predominantly have a Leu residue and sometimes Phe, and low redox potential laccases have a Met residue [31]. This observation was confirmed using site-specific mutagenesis [39,62,63,64]. In addition, the redox potential of the T1 center of laccases depends on the composition and structure of the loops around the Cu1 copper center [65,66,67]; laccases which have a higher number of large hydrophobic residues (e.g., Phe) in the vicinity of the Cu1 copper ion that cover it from the solvent are characterized by a higher redox potential [34].

**Figure 5:**
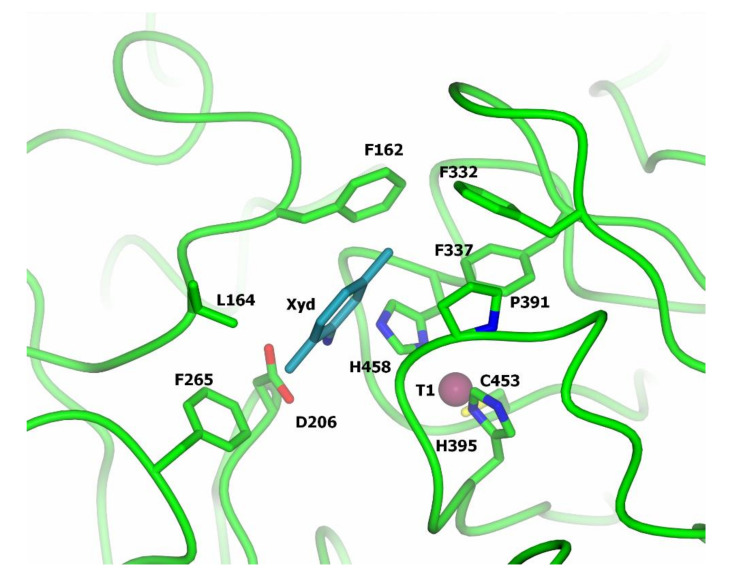
Substrate-binding pocket of *T. versicolor* laccase (structure of the laccase complex with 2,5-xylidine, PDB 1KYA [68].

For bacterial and fungal laccases, a number of structures of laccase complexes with electron donor substrates were determined [65,66,67,68,69,70,71] which allowed the highlighting of the loops that form the substrate-binding pocket. In three-domain laccases, the substrate-binding pocket for oxidizable phenolic compounds was found to be formed by the loops of domains I and II located near the T1 center (Figure 5). The substrate-binding pocket is formed mainly by hydrophobic amino acid residues (Figure 5, residues Phe162, Leu164, Phe265, Phe332, Phe337, Pro391); it also includes the His458 residue coordinating the Cu1 copper ion. Presumably, a polar residue at position Asp206 is also involved in substrate binding (Figure 5). It should be noted, however, that the resolution of the structures of the complexes of laccases with substrates was not high and the position of the substrate molecule was different for different laccases and different substrates, which indicates non-specific binding. Moreover, the amino acid composition of the loops of the substrate-binding pocket varies greatly among laccases from different organisms [34], which makes it difficult to predict the binding mode of the substrate. In addition, it was shown that the more flexible substrate-binding pocket loops can also facilitate the oxidation of bulky substrates with a rigid structure despite the redox potential value of the laccases [28].

**Figure 6:**
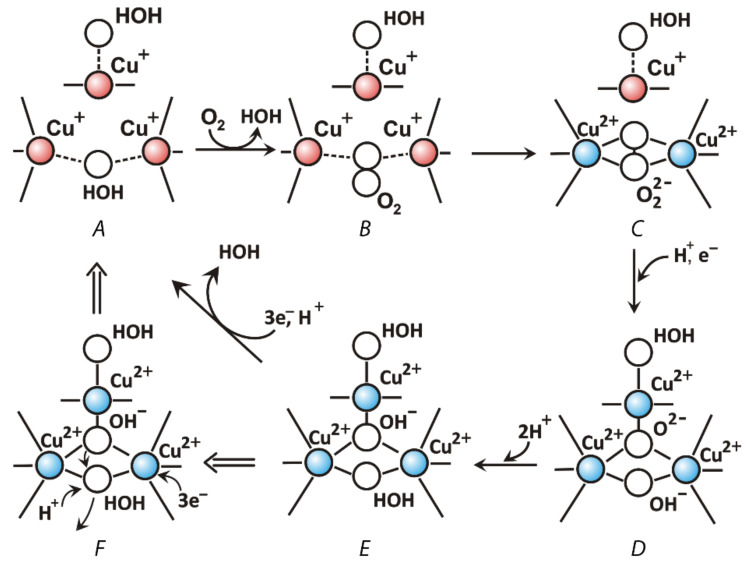
Mechanism of molecular oxygen reduction by laccases to water: O_2_ + 4e^−^ + 4H^+^ → 2H_2_O. Intermediate states are shown in Figure (**A**–**F**). Panel F shows the mechanism of the release of a water molecule from the TNC. Coordination and covalent bonds are shown by solid lines. Ion-dipole electrostatic interactions are shown with dotted lines.

As it was mentioned before, there are three copper ions in the TNC where the molecular oxygen is reduced by laccase. The T2 copper ion is coordinated by side chains of two histidine residues and T3 copper ions—by three histidine residues each (Figure 3). TNC can be reached by water and protons from the reaction medium through the T3 and T2 water channels. The mechanism of oxygen reduction to water by laccase was originally proposed by Solomon et al. on the basis of spectroscopic data and the three-dimensional structure of a laccase-related enzyme, ascorbate oxidase [72]; later, it was confirmed [73] and detailed [74] by the X-ray diffraction analysis. Modern views on the mechanism of oxygen reduction to water by laccases are summarized in the review [25] and updated with X-ray diffraction data in [74]. The oxygen reduction process consists of two stages of two-electron reduction processes. The catalytic cycle begins with a fully reduced enzyme (all copper ions are in the +1 oxidation state, Figure 6A) with two oxygen ligands (W2 and W3, Figure 4) that are bound to copper ions of TNC. The Cu2 copper ion forms a bond with the W2 oxygen ligand that is located outside the T2/T3 center. When a chloride or fluoride ion binds at this position, laccase inhibition takes place [75]. The W3 is located between the pair of Cu3 ions and can be substituted by molecular oxygen (Figure 6A, and 6B). Then, during the first two-electron reduction, molecular oxygen oxidizes both copper ions to the +2 oxidation state and becomes a deprotonated peroxide molecule (first two-electron reduction, Figure 6C). It can be protonated from the side of the T3 water channel. The second two-electron reduction process involves the electron transfer to peroxide from the Cu2 copper ion and from the Cu1 copper ion (via the Cu3 copper ion, Figure 6D). As a result, an O-O bond in the peroxide is cleaved and Cu1 and Cu2 ions become oxidized (+2). Subsequent protonation of oxygen ligands leads to the formation of a hydroxide ion and a water molecule (Figure 6E). The release of oxygen ligands from the TNC takes place due to the reduction of copper ions of the T2/T3 center by electrons that are transferred from the Cu1 copper ion (Figure 6F). The OH^−^ ligand at position W1 replaces the W3 water molecule which leaves the active site. The OH^−^ ligand can be protonated and become a water molecule. Thus, the system returns to its original state (Figure 6A).

### 2.4. Laccase Industrial Application

LCs have become relevant industrial enzymes due to their broad range of activity towards phenolic and non-phenolic compounds. As recently reviewed by Moreno et al. [76], many industrial applications can be performed by LC, as well as in combination with redox mediators, especially for recalcitrant compounds. From the industrial perspective, LC mediators should be environmentally friendly and cheap. Currently, the most effective mediators are chemically synthesized compounds, however, they are usually hazardous and quite expensive to produce [77]. This leads to the development of alternative uses of naturally-occurring phenolic mediators originating from lignin biodegradation or from vascular plants either as extractives or lignin-forming monomers [78].

In textile and paper pulp, fungal LC is used for bio-bleaching, i.e., the degradation of synthetic dyes and inks, which are chemically heterogeneous chromogenic compounds. They can be toxic and genotoxic, and, once released into water bodies, they reduce the oxygen content and the photosynthetic activity of microorganisms [78]. Due to their synthetic origin and complex aromatic structures, they are recalcitrant to the conventional microbial degradation or decolorization. LC is able to degrade them but often in combination with the synthetic mediators hydroxybenzotriazole (HBT), ABTS and violuric acid (VA) [8,12,79].

In biorefinery, LC application has different purposes: (i) delignification, (ii) detoxification, i.e., reduction of the phenolic content, and (iii) reducing the adsorption of hydrolytic enzymes to lignin. Delignification is a mandatory step to make carbohydrates more accessible to carbohydratases in the subsequent saccharification process. Nonetheless, phenols can be released after enzymatic and chemical delignification as well as steam explosion. Phenols act as inhibitors of enzymes and fermentative yeasts, thus a detoxification process may be needed to boost both the saccharification and fermentation yields [76].

In food applications, LC is used to remove the phenols that are responsible for turbidity and color instability in wine, beer, and juices [80,81]. Alternatively, LC can catalyze the formation of crosslinks between proteins, also by oxidizing natural mediators, which act as crosslinking agents [82]. Crosslinks in food matrix generated by LC allow to modify the rheological, technological, and nutritional properties of vegetables, cereals, and milk-based products, thus creating novel foods with improved characteristics [83,84,85,86].

Bioremediation by LC encompasses the oxidation of phenols from industrial effluents, dyes, and toxic compounds, such as mycotoxins, polycyclic aromatic hydrocarbons, endocrine disruptors, and antibiotics [86,87,88,89]. Also in this case, redox mediators are often needed to ensure an effective level of detoxification.

Laccase use in organic synthesis has been increasing in recent years. Substrates such as substituted phenols, polyamines, anilines, aromatic and alkyl amines, and benzenethiol are the starting point for biocatalytic reactions for the syntheses of bioactive compounds for pharmaceutical and nutraceutical applications [90]. The synthesis of new compounds may include simple oxidation/amination/thiolation reactions and oxidative coupling reactions. Homomolecular coupling can be used to produce compounds that are endowed with new or improved biological activities. LC-mediated coupling was used on the simple phenols, such as 2,6-dimethoxyphenol to obtain compounds with increased antioxidant activity; on complex phenolic compounds, such as rutin, to improve their solubility in water; or resveratrol, to produce compounds with antiproliferative activity towards colon cancer cells [91] or new antibiotics (e.g., aminopenicillins, aminocephalosporins, aminocar-bacephems, and sulfonamide derivatives). Eventually, LC- mediated homomolecular coupling was also exploited to reduce the concentration of several water pollutants including estradiol, triclosan, and cumylphenol [92,93,94].

Grafting, e.g., coupling reactions involving a natural or synthetic polymer, can be used to functionalize polymers to confer new features. LC-mediated grafting has multiple applications, including environmental pollution control, modification of lignocellulosic material/foods/ingredients in textile industry, biosensors, food industry, pharmaceutical industry, and organic synthesis [95].

LCs, together with several other oxidoreductases, have found applications in the construction of enzymatic biosensors and bio-cells for biomedical, food safety, and environmental monitoring purposes. While in the classic biosensors, LC is immobilized directly on an electrode, in bio-cells the enzyme are displayed on the membrane of the whole microorganism that is grown on an electrode [96]. The advantages rely on the longer lifetime due to the possibility of the microorganisms duplicating and creating a biofilm on the electrode [97].

## 3. Re-Use of Agro-Wastes for Laccase Production

### 3.1. Laccase Production by Solid State Fermentation Using Agro-Wastes

Enzymes can be produced by two different approaches: solid state (SSF) and submerged fermentation (SmF) [6]. SSF is performed on a heterogeneous, porous, low moisture, solid material, where microorganisms are able to grow whether on or within the surface material. On the contrary, SmF is performed in a liquid medium in which optimized nutrients are dissolved.

SSF holds a great potential for LC production by filamentous fungi, as they are able to colonize the substrate and promote the hyphal growth on or in the particle. SSF mimics the natural fungal environment and naturally contains LC inducers, such as flavonoids and phenols. This is a great advantage because the use of inducers, such as metal ions (copper and, to a lesser extent, manganese), synthetic compounds such as ABTS, or ethanol in SmF is often needed to achieve satisfactory enzyme yield and poses both economic and environmental concerns. [98,99].

SSF allows the easy production of extracellular enzymes, has lower energy requirements, lower environmental impact, lower risk of contamination, easier downstream processing, and higher volumetric and enzyme yield than SmF [100].

White-rot fungi are among the best LC producers because they naturally use LC together with peroxidases to degrade lignin and access the plant polysaccharides which are then used as carbon source [101]. Lignin is the structural component of plants and it is responsible for their impermeability, resistance towards microbial attacks, and oxidative stress. Owing to its resistance, it represents a major obstacle to reconvert agro-wastes into valuable compounds [102]. Lignin is also recalcitrant to mild chemical and biological degradation techniques and it can be efficiently degraded only by microorganisms which possess a wide array of oxidative enzymes. White-rot fungi are key players in the global carbon cycling and among the main producers of LCs and peroxidases which enable them to degrade this recalcitrant biopolymer [96,103,104].

The carbon to nitrogen (C/N) ratio is a crucial parameter for fungal growth by SSF, as it influences the mycelial growth and the production of several compounds. The optimal C/N ratio has to be assessed for each fungal species, although higher C/N ratios (30–40) have been reported to promote the fastest mycelial growth (colonization rate), fruiting bodies, and polysaccharide production, while lower values (10–20) were reported to give the highest overall biomass and LC production [98,99]. To balance C/N ratio, ammonia or organic sources, such as yeast extract, can be added to significantly increase LC production [105].

The reactor type and airflow system are also important determinants for the fungal growth because they determine how the air, containing O_2_, is provided; how the correct moisture is maintained; and how the metabolic heat and the CO_2_ are dissipated [106].

Using agro-wastes for SSF allows the use of cost effective resources to reduce the environmental impact of their disposal and to boost LC yield thanks to the presence of inducers [93]. Spent grains, straw and residues of cereal harvesting, fruit peels, and general food waste are perfect examples of economic and sustainable ingredients for LC production by SSF; i.e., this material can be further exploited to produce energy. The most recent studies on LC production by solid state fermentation (SSF) on sustainable substrates are reported in Table 1.

Brewers’ spent grains (BSG) are the solid by-product of beer production and they are usually disposed as cattle feed. Nonetheless, they can be used as material for LC production by SSF. Additionally, lignin degradation by fungi can drastically enhance polyphenol recovery from lignocellulosic biomass [100,107].

SSF with BSG was performed with *T. versicolor*, obtaining a maximum LC production of 560 U/L after 7 days and 3.4-fold increase in the extraction of total polyphenols [108]. Dhillon et al. used BSG that was supplemented with different inducers. Despite the fact that they obtained good results without inducers (up to 2343 IU/g dry weight in flasks and 2956 I U/g dry weight in trays), the addition of different compounds significantly increased LC production. The best one was phenol (10,108 IU/g dry weight in flasks and 13,506 IU/g dry weight in trays) [109].

Different agro-wastes, specifically wheat- (WS) and bean-straw (BS) and reed grass (RG), were used as main ingredients for the production of LC by SSF with three strains of *Lentinula edodes*. In this study, lower lignin content and higher cellulose content gave the best results in terms of LC production and timing. WS was the best material, as it led to 579 U/g per dry weight after 25 days of cultivation [110]. Corn stalks were used for *T. versicolor* cultivation as raw materials and after a steam explosion. The pre-treatment was able to increase LC production by 2.1-fold (up to 2600 U/g after 15 days), likely as a result of polyphenol that was released from lignin (from 1.98 to 3.54 mg of gallic acid equivalent per g) and the increased accessibility of polysaccharides [111]. Similarly, rice straw was used raw and after a chemical pre-treatment with ammonia for LC production by *Funalia trogii*. Also in this case, the pretreatment boosted LC production up to 3.4 times due to the modification of the chemical composition of the rice straw [112]. When different pretreatments with ammonia were used, the harshest one, leading to the highest cellulose content, gave the highest LC yield [112]. The importance of the type of lignocellulose substrate in inducing LC was proven also in the work of Patel and Gupte [113]. They compared different raw materials for the production of LC by the fungus *Tricholoma giganteum*, namely wheat bran, wheat straw, rice bran, and rice straw. Wheat bran was the best material for fungal growth and LC production, which was increased by several fold (up to 89,800 U/g) under optimized conditions and by using copper sulphate and phenolic inducers.

*Coriolus versicolor* was cultivated in a mesh tray bioreactor using sorghum bagasse to produce different ligninolytic enzymes, including LC. The optimization of mesh sizes in the tray and the airflow rate led to a 1.9-fold increase in LC production [114], confirming the important effect of the reactor type and airflow system for the fungal growth and enzyme production.

Tea residues deriving from tea beverages, instant tea production, and polyphenol extraction can be used as a substrate for fungal growth and LC production, as described by Xu et al. [115]. Compared to the cereal derived wastes, the tea residues showed lower lignin and cellulose content in favor of higher hemicellulose. Nonetheless, the authors were able to produce up to 31.2 U/g of LC after only eight days. A different approach was used by Pourkhali et al., who isolated an LC-producing fungi from the olive oil mill wastewater to produce LC using the olive-leaf wastes [116]. In this way, they were able to use an already adapted fungal species and boosted LC production thanks to the addition of another waste, wheat straw. In this work, the reaction parameters were also optimized by the Taguchi method of an orthogonal array design of experiment (DOE) to obtain up to 56 U/g of LC after 14 days of fermentation.

These recent studies demonstrate that using sustainable substrate for LC production is feasible and allows the valorization of different kinds of wastes and reduce the costs of LC production and the environmental pollution that is derived from their disposal. Pretreatments, redox mediators, and parameter optimization are often needed to boost LC production or increase the rate of production.

Scaling up of LC production by SSF, enzyme immobilization, and purification technologies remain future activities to investigate for successful and simple industrial application.

### 3.2. Laccase Immobilization on Agro Industrial Wastes

Enzyme immobilization is defined as a process which binds a soluble enzyme to a carrier matrix to generate an insoluble species with improved catalysis and resistance to harsh environmental conditions [117]. Immobilization has been widely used in industry to enhance enzyme stability and its recovery in batch or continuous reactor operations [118,119]. The attachment to a matrix makes the enzymes insoluble and thus, easier to recover, and improves the general stability by making the enzyme more rigid, delaying its degradation or unfolding, and preventing the inhibition by the substrate [120].

Nowadays, increasing attention is given to carrier materials which are cost-effective, safe, biodegradable, and environmentally friendly. Together with the traditional biobased immobilization materials (agarose, starch, chitosan, alginate), agro-industrial wastes represent an important opportunity to improve circularity and sustainability in the food system [121]. Lignocellulosic waste, spent grains, eggshell membrane, biochars, and chicken feathers were successfully used to immobilize LCs, as well as other enzymes from fungal origin [122], as reported in Table 2.

Spent grains (SGs) are a waste that is generated after the mashing and lautering of beer. Due to the presence of many functional groups (carboxyl, hydroxyl, and amino) to which LC can be covalently linked or adsorbed, it can be used as a carrier for enzyme immobilization. da Silva et al. (2012) immobilized a commercial LC from *Aspergillus* spp. that is used in textile industry by covalent bonding on SGs to obtain higher storage, operational, and thermal stability [123]. Similarly, Girelli and Couto (2021) immobilized a commercial LC preparation from *T. versicolor* by adsorption, although this resultd in a significantly lower catalytic activity [124].

Eggshell membrane (ESM) is a by-product of egg transformation chain and represents an interesting immobilization support for LC immobilization. ESM is mainly composed of proteins (80%) as well as being a cross-linked, water insoluble, and a porous material. The main protein components are collagen, hyaluronic acid, and glucosamine, which are rich in amino, hydroxyl, carboxyl, and thiol groups that are needed for immobilization [125]. A commercial LC from *T. versicolor* was immobilized in ESM by means of both covalent bonding and adsorption [126]. In this preliminary study, different protocols were compared and the reusability was 30–40% after six cycles.

Immobilization of LC on coconut fibers (CFs) has been widely explored for LC in both food and non-food applications. Adsorption of a commercial LC from *T. versicolor* on CFs proved to extend the stability and reuse of LC over 10 cycles for fruit juice clarification [127]. Cristóvão et al. investigated two different immobilization techniques for a commercial LC from *Aspergillus* spp., namely adsorption and covalent bonding on CFs. Covalent bonding proved to be more efficient than adsorption to increase operational and storage stability, as well as activity towards textile dyes [128,129]. Nonetheless, adsorption of the dyes to the carrier was observed so reversibility of the reaction should be taken into account in further studies.

Biochar is the product that remains after the pyrolysis of a biomass, i.e., burning in the absence of or with limited air to temperatures of over 250 °C. Due to its chemical stability, high surface area, and porosity, it has been used as a carrier for enzyme immobilization, mostly by adsorption. With regard to LC immobilization, different studies were performed to remove environmental pollutants. Li et al. immobilized an LC from *Coprinus comatus* on two different wood biochars deriving from maple and spruce, obtaining the best results with maple biochar that had a higher surface area and pore volume. A higher stability, reusability (five to seven cycles) and enhanced enzymatic degradation towards chlorinated biphenyls were obtained [130]. Rice straw biochar was also used to immobilize an LC from *T. maxima* for an effective anthracene degradation [131]. The enzyme retained 66% of activity after immobilization and up to 60% activity was retained after six cycles of operational use.

Covalent bonding of an LC from *T. versicolor* to pine wood, pig manure, and almond shell biochars was also tested for diclofenac removal [132]. As reported in other studies, the most promising results were obtained for biochars with a higher surface area. In addition, functionalization with citric acid was shown to improve LC covalent immobilization up to 20%.

Chicken feathers are a waste by-product of the poultry industry. Common disposal methods are harmful for the environment and include incineration or burial in landfills [133]. Suman et al. [134] covalently immobilized a LC from *T. maxima* with chicken feathers that were functionalized with amino 3-aminopropyltrimethoxysilane. They obtained a LC with higher thermal stability and no significant loss of enzyme activity after eight cycles. The complete degradation of veratril alcohol was obtained in 48 h.

Compared to the use of synthetic carriers, the use of agro-wastes reduces the environmental impact of enzyme immobilization. Nonetheless, in most cases the treatment with chemicals or functionalizing agents is needed [122].

These processes have been validated only at the laboratory level, thus further studies are needed to assess scale-up and efficacy in industrial operational conditions. An important issue that needs to be addressed in operational conditions is the possible enzyme leaking from the carrier material, which has to be evaluated also in accordance with the final use of the treated material (food, feed, raw materials for fermentation etc.). When using high performing enzymes, it is mandatory to use low dosages and reduce the risk of having high enzyme amounts being possibly released from the carrier. Nonetheless, the studies that were presented here showed promising results also in terms of enzyme leakage after multiple cycles.

A further step towards sustainability, scale-up, and economic feasibility has to be done to reduce the use of these chemicals that are needed to pretreat the material. Despite that, the impact of the disposal of such wastes, generally by burning, still remains more concerning.

### 3.3. Valorizarion of Agro-Wastes by Laccase Pretreatment

As already mentioned, biorefinery for energy production is one of the main pathways that LC pretreated agro-wastes may enter as raw materials. Indeed, the so-called second-generation bioethanol employs lignocellulosic biomass and wastes as a source of fermentable sugars [135]. Nonetheless, the pretreatment to remove or degrade lignin is mandatory. Effective delignification increases sugar accessibility and enhances the following saccharification step, while the removal of phenols allows the microorganisms to efficiently ferment and produce bioethanol or methane. Purified LC preparations were successfully used for the delignification of different vegetable biomasses (Table 3).

A purified LC from *Trichoderma asperellum* was used to pretreat sweet sorgum stover [136]. Thanks to its high stability at high temperatures and low pH, the authors were able to remove of up to 77% of lignin and obtain a 3.26-fold increase in biohydrogen production, proving that this LC could represent an important tool to be used in biofuels conversion. Giacobbe et al. [137] used two different LC preparations from *P. ostreatus* to de-lignify three different agro-wastes, namely apple pomace, potato peels, and coffee silverskin. The treatments with and without the addition of vanillin as a redox mediator were effective in reducing the lignin content (73 and 83%, respectively), to remove phenols, and to obtain satisfactory saccharification yields. The authors highlight that using such enzymatic pretreatment allows the avoidance of filtration and washing steps, with important advantages in sugar retention for the subsequent saccharification step and wastewater production [136].

Pineapple leaves are an important agro-industrial waste which can be valorized for biofuel production after lignin removal. Banerjee et al. treated this waste with an LC preparation from *P. djamor* with optimized solid loading (20%, *w*/*v*), incubation time (six hours), temperature (40 °C), pH (7), and enzyme concentration (500 IU/mL) obtaining the removal of up to 78.57% of the lignin and a 2.6-fold increase in reducing sugars [138]. Similarly, Sherpa et al. optimized the sugarcane top pretreatment with an LC from *P. djamor*. In accordance with the study of Banerjee et al., they obtained optimized conditions for solid loading (21%, *w*/*v*), incubation time (six hours), temperature (40 °C), pH (7), and enzyme concentration (430 IU/mL) obtaining the removal of up to 79.1% of lignin and a 3.3-fold increase in reducing sugars [139].

The redox mediator HBT was used in combination with an LC from *P. cinnabarinus* for the pretreatment of sugarcane bagasse and straw, with the aim of removing lignin and reuse these wastes for energy production [140]. Thanks to the LC treatment, the lignin was reduced by 27% and 31% from sugarcane bagasse and straw, respectively, allowing the release of 39% and 46% of glucose for the saccharification step.

*T. maxima* LC was used in combination with HBT to pretreat the jute sticks, a widely abundant waste that is derived from jute fiber extraction [141]. By using the laccase-mediator treatment, the lignin was reduced up to 21.8% with a single reaction and the yield of fermentable sugars was increased by 19.5%.

Rajac and Banerjee studied the delignification of kans grass for bioethanol production in two different studies using different conditions for the enzymatic hydrolysis [142,143]. Promising results in terms of delignification, sugar release (up to 500 mg/g), and bioethanol production (increase of 9%) were obtained.

LC pre-treatment of vegetable biomass can be also helpful for the production of sustainable compost. Besides being hardly degraded by the microorganisms that are used for composting, lignin hinders humus formation. The addition of lignin-degrading microorganisms has been proven to boost the rate of composting, its maturity, stability, and final quality [145]. The use of whole microorganisms is widely spread, especially because the spent mushroom substrates can be used for composting, as fertilizers, or for remediation purposes [146]. So far, only the use of an LC preparation from *T. versilocolor* was reported by Nazilah et al., who treated the coffee bean processing waste to improve its composting quality. The reduction in lignin and phenols allowed it to have a higher and more stable microbial count and to improve the compost quality [146].

The use of enzymes in industry is a reality in many fields of application. In particular in biorefinery processes, cellulases have been mostly applied to recover the simple sugars from the polysaccharide fraction. Therefore, the implementation of the use of an LC for the pretreatment of the vegetable biomass is feasible. Nonetheless, to save costs, reduce the environmental impact, and maximize yields, it requires a careful analysis of technical solutions that enable the design of a single reactor to perform delignification, saccharification, and fermentation in a continuous or sequential mode [144]. Eventually, it is important to study the delignification kinetic and the enzyme stability/activity/adsorption to the lignocellulosic material in a bioreactor to obtain reproducible and satisfactory results also when material with different lignin/cellulose/hemicellulose ratio is used.

## 4. New Trends and Challenges for Laccase Application

### 4.1. Cold-Active Laccases

A novel trend in enzyme technology is the use of cold-active enzymes, namely extremozymes that are produced by psychrophiles which have a high catalytic activity at moderate-low temperature [147]. The use of such enzymes is of particular interest because they allow the treatment of agro-wastes and vegetable biomass in the site of production. This is because the process can be performed at room temperature, saving the energy that is required to heat the biomasses as well as without complex transportation procedures.

Moreover, such enzymes can be used to process heat-sensitive products in the food industry. LCs for phenolic compounds removal in chilled juices, wine, and ready to drink coffee and tea, or to improve the texture in surimi, yogurt and ice cream, and meat represent possible applications of cold-active LC in the food industry [148]. Eventually, the thermolability of cold-active enzymes will allow an easy control of the process since they can be selectively inactivated by mild heat [148].

The interest in cold-active and cold-adapted LCs has risen, in particular for the possibility of removing contaminants from effluents or wastes that are directly in the site of contamination or without the need for elaborate equipment. Table 4 summarizes the most recent studies on cold-active LCs.

Shi et al. [149] isolated a cold-active LC from *Botrytis* sp. FQ grown on tomato fruits that were stored at 4 °C. The enzyme showed 70% activity at temperatures between 0 °C and 30 °C and an optimal temperature of 15 °C on 2,6-dimethoxyphenol, a classic LC substrate. When tested in operational conditions, pH 6.8, 20 °C, the LC was able to remove up to 60% of triclosan. A cold-adapted LC from *Kabatiella bupleuri* was isolated and characterized by Wisniewska et al. [150]. The enzyme had an optimal temperature of 30 °C. Nonetheless it retained 60% of its maximum activity at 10 °C and over 40% in ice. Interestingly, the LC could be efficiently produced using a sustainable material (BSG) such as a carbon source in SSF and used for dye decolorization. Lac-Q from *Pycnoporus* spp. showed an optimum temperature of 70 °C but retained about 51% of the maximal activity when incubation in ice. Lac-Q was used in combination with ABTS to degrade oxytetracycline [151].

### 4.2. Alkaline Active Laccase

LCs are generally stable at alkaline pHs. Nonetheless at such pHs, their activity is barely detectable [152]. This pH profile of most fungal LCs was explained by an inhibitory effect of the OH- ions on the T2 and T3 centers, as well as by the redox potential difference between the reducing substrate and the T1 center at an alkaline pH [154]. Alkaline-active LCs are rare and can be found in alkaliphilic organisms or obtained by protein engineering. In the latter case, structure-based amino acid substitution near the T1 and TNC sites proved to be effective in shifting LC optimum pH activity [152].

Catalytic activity at a high pH is a desirable characteristic in certain industrial contexts such as bioethanol production, hair coloring, paper bio-bleaching, or pollutant removal from wastewaters. Lignin is insoluble at neutral-acidic pHs thus, an LC could be applied for its depolymerization only after solubilization in alkaline solution [155]. Wastewaters from textile industries generally possess a neutral to alkaline pH (7–10) because dye bleaching is traditionally performed using an alkali solution, such as sodium hypochlorite [156]. Therefore, the study of alkaline active LCs which could be potentially used in such industrial applications is an important topic of research that has been explored little (Table 4).

Protein engineering via error-prone PCR was used to obtain LC mutants which exhibited high activity towards 2,6-dimethoxyphenol at pH 8–8.5. When tested in operational conditions (pH 7.0–7.5) it efficiently decolorized 87% of indigo dye [153]. A knowledge-gaining directed evolution (KnowVolution) process was used to increase the alkaline tolerance of an LC from *Melanocarpus albomyces*. By means of two single mutations near the T1 site, the authors were able to obtain an LC with an optimum pH of 9 for the substrate 2,6-dimethoxyphenol [157]. Eventually, directed evolution was used to obtain a blood tolerant LC for LC-based bio-electronic devices application [158]. By introducing mutations near the T1 site, the authors were able to shift the pH activity profile of a thermostable LC to more alkaline values and to significantly enhance NaCl tolerance.

The research on these topics is still at an early age and further studies are needed to ensure the implementation of enzyme production at industrial level, enzyme improvement to avoid the use of synthetic mediators, and the toxicological assessment of the degradation products in the case of remediation applications.

The search for an LC with extended catalytic properties, stability, and activity in the harsh environmental conditions that are typical of industrial processes is a major topic of research. As shown by the studies reported here on cold- and alkaline-active LCs, extremozymes can be found in nature, but most likely can be improved or created in the laboratories thanks to the application of biotechnology.

Rational and random mutagenesis by error-prone PCR has represented the method of choice to improve LC catalytic activity in the past. Later, other techniques, such as site-directed mutagenesis, semi rational design, and directed evolution have been used and often combined in a stepwise mutagenesis approach [158]. Recently, DNA shuffling, directed evolution, site-directed, and site-saturation mutagenesis have been applied to obtain engineered, tailor-made LCs or chimeras with increased thermostability and activity under harsh environmental conditions (extreme temperatures, pHs, presence of surfactants, solvents, or enzyme inhibitors). Moreover, the use of computational methods to flank protein engineering is opening new opportunities to deliver tailored catalysts for specific applications [158].

## 5. Conclusions

Enzymatic catalysis is an essential pillar to ensure sustainability in industrial processing. Enzymes are safe, selective, specific, biodegradable, and require mild operational conditions. Nonetheless, their application in an industrial context is limited due to difficulties in scale-up, discouraging technology readiness, and process economics. Among the many industrial enzymes, LCs show a unique profile in terms of greenness, versatility, pollutants removal, and efficiency in lignin degradation for the exploitation and valorization of agro-wastes. Indeed, lignin removal is one of the main limitations in closing the loop of agricultural systems because it is the main obstacle for the reuse of wastes as raw materials for the production of valuable compounds or for fermentations. Nonetheless, in recent decades, research has made many steps forward to fully exploit this important enzyme, ensure the economic feasibility of its production, and to overcome the technical limitations of its application. In this review the most recent advances for LC production, immobilization, and use of ago-wastes were given. At the laboratory level these methods proved to be efficient and feasible. Nonetheless, a continuous crosstalk and collaboration with industries is needed to let the new era of LC-sustainable use in industry begin.

Still, after over 100 years from its discovery, LCs remain one of the most promising green enzymes for sustainability, reuse, valorization, and decontamination of vegetable biomasses.

## Figures and Tables

**Table 1:** Laccase production by solid state fermentation (SSF) on sustainable substrates.

SSF Substrate	Species	Substrate Composition and Growth Parameters	LC Activity	Reference
Brewers’ spent grains (BSG)	*Trametes versicolor*	Lignin 8.53%, cellulose 16.1%, hemicellulose 20%, ash 5.3% DW. Growth at 27 °C for 14 days	560 U/L after 7 days	[108]
BSG added with LC inducers	*T. versicolor*	Lignin 12.4%, cellulose 13.8%, hemicellulose 30%, ash 2.6% DW. Growth at 30 °C for 16 days	13,506 IU/g using 10 mg/kg phenol as inducer after 12 days	[109]
Wheat straw (WS), bean stalk (BS), and red grass (RG)	*Lentinula edodes*	WS: Lignin 7.58%, cellulose 68.93%, hemicellulose 11.16% DW.	579 U/g DW after 25 days	[110]
		BS: Lignin 11.27%, cellulose 64.65%, hemicellulose 11.27% DW.	258 U/g DW after 25 days	
		RG: Lignin 7.5%, cellulose 69.22%, hemicellulose 10.69% DW. Growth at 26 °C for 40 days	390 U/g DW after 35 days	
Corn stalk (raw and steam exploded)	*T. versicolor*	Lignin 22.43%, cellulose 33.96%, hemicellulose 13.95% DW. Growth at 30 °C for 7 days	2600 U/g after 15 days	[111]
Rice straw (raw and ammonia-treated)	*Funalia trogii*	-	172 U/g after 14 days	[112]
Wheat and rice straw and bran	*Tricholoma giganteum*	- Growth at 30 °C for 20 days	89,800 U/g after 16 days	[113]
Sorghum Bagasse	*Coriolus versicolor*	Lignin 25.14%, cellulose 38.02%, hemicellulose 25.01% DW. Growth at 27.5 °C for 20 days	115 U/g after 20 days	[114]
Tea residues	*T. versicolor*	Lignin 13.60%, cellulose 11.60%, hemicellulose 32.50% DW. Growth at 26 °C for 8 days	31.2 U/g after 8 days	[115]
Olive leaves and wheat straw	*Galactomyces geotrichum*	- Growth at 30 °C for 26 days	56 U/g after 14 days	[116]

**Table 2:** Laccase immobilization on sustainable carriers.

Carrier Material	LC Origin	Immobilization Method	Results	Application	Reference
Brewer’s spent grain	Commercial LC (DeniLite base) from *Aspergillus* spp.	Adsorption to acid/base treated spent grain	Recovered activity 99%, immobilization yield 95%, 75% activity retained after 10 cycles at pH 10	-	[123]
Spent grains	Commercial LC (S igma-Aldrich) from *T. versicolor*	Imine binding with acid/base treated spent grain and adsorption to spent grain	Recovered activity 39%, immobilization yield 1.3%, 58% activity retained after 6 cycles	58% removal of syringic acid after 6 cycles and complete removal in 4 h	[124]
Egg shell membrane	Commercial LC (S igma-Aldrich) from *T. versicolor*	Covalent bonding and adsorption	Recovered activity 57%, immobilization yield 41%, 40% activity retained after 6 cycles	57% removal of syringic acid after 24 h	[126]
Green Coconut fibre (CF)	Commercial LC (DeniLite base) from *Aspergillus* spp.	Acid/oxidative pretreatment; Covalent attachment to functionalized CF	Immobilization yield 74%, 55% activity retained after 10 cycles	Up to 70% degradation of textile dyes. Operational activity is significantly reduced in following cycles.	[127]
CF	*T. versicolor*	CF was subjected to acid/base pretreatment associated with thermal decompression. Adsorption to functionalized CF; covalent bonding via glutaradehyde.	Recovered activity 59%, immobilization yield 98%, 16.5-fold increase in thermal stability, 80% activity retained after 10 cycles in operational conditions	65% reduction of phenolic compounds from apple juice	[129]
Maple biochar	*Coprinus comatus*	Adsorption to maple biochar	Recovered activity 66.5%, immobilization yield 64%, 66% activity retained after 7 cycles.	71.4% degradation of chlorinated biphenyls after 5 h of treatment (2.5 times higher than the free enzyme)	[130]
Rice straw biochar	*T. maxima*	Adsorption to acid-treated rice straw biochar	Recovered activity 66%, immobilization yield 100% at pH 3.1, 60% activity retained after 6 cycles	98% degradation of anthracene after 24 h (comparable to the free enzyme)	[131]
Pine wood biochar	*T. versicolor*	Covalent immobilization via glutaraldehyde to citric acid pretreated biochars	Recovered activity 20.1%, 46% activity retained after 5 cycles	98.9% removal of diclofenac after 5 h of treatment	[132]
Pig manure biochar			Recovered activity 40.2%, 40% activity retained after 5 cycles	98.9% removal of diclofenac after 2 h of treatment	
Almond shell biochar			Recovered activity 31.8%, 43% activity retained after 5 cycles	98.9% removal of diclofenac after 4 h of treatment	
Chicken feathers (Chf)	*T. maxima*	Covalent immobilization via glutaraldehyde to Chf functionalized with amino 3- aminopropyltrimethoxysilane	Recovered activity up to 93%, immobilization yield up to 74.24%, no significant loss of activity after 8 cycles	Complete oxidation of veratryl alcohol after 48 h	[134]

**Table 3:** Laccase application for the valorization of agro-wastes.

LC Origin	Application	Results	Reference
*Trichoderma asperellum*	Pretreatment of sweet sorghum stover for biohydrogen production	Removal of up to 77% of lignin; 3.26-fold increase in biohydrogen production.	[136]
*Pleurotus ostreatus*	Pretreatment of apple pomace, potato peels, and coffee silverskin for energy production	Up to 83% saccharification yields and ∼70% phenol reduction using 2.5% vanillin as redox mediator	[137]
*P. djamor*	Valorization of pineapple leaf waste for biofuel production	Removal of up to 78.57% of lignin, 2.6-fold increase in reducing sugars	[138]
*P. djamor*	Pretreatment of sugarcane tops	Decrease of up to 79.1% of lignin content; increase of 3.3 fold in fermentable sugars	[139]
*Pycnoporus cinnabarinus*	Valorization of sugarcane bagasse for energy production	Decrease of up to 27% of lignin content; increase of 39% of glucose release	[140]
	Valorization of straw for energy production	Decrease of up to 31% of lignin content; increase of 46% of glucose release	
*Trametes maxima*	Pretreatment of jute sticks for energy production	Decrease of up to 21.8% of lignin content; increase of 19.5% of glucose release using 5% HBT as redox mediator	[141]
*Lentinus squarrosulus* MR13	Pretreatment of kans grass (Saccharum spontaneum)	Decrease of up to 87.8% of lignin content; increase of 9% of bioethanol production	[142]
		Decrease of up to 81.2% of lignin content; production of up to 500 mg/g of fermentable sugars	[143]
*T. versicolor*	Pretreatment of coffee bean processing waste for composting	Increase in total plate count values	[144]

**Table 4:** Cold-active and alkaline active laccases and their application.

LC origin	Application	Results	Reference
*Botrytis* sp. FQ	Removal of antibiotics	70% activity at temperatures between 0 °C and 30 °C and an optimal temperature of 15 °C on dimethoxyphenol and removal of up to 60% of triclosan at pH 6.8, 20 °C	[149]
*Kabatiella bupleuri*	Dye decolorization	Retention of 60% of the maximum activity at 10 °C and over 40% in ice; up to 48.6% degradation of crystal violet after 1-h reaction with ABTS	[150]
Lac-Q from *Pycnoporus* spp.	Removal of antibiotics	Retention of 51% of the maximum activity at in ice; degradation of 50 mg L^−1^ of oxytetracycline at pH 6.0 and 0 °C after 5 min with ABTS	[151]
*Melanocarpus albomyces*	Oxidation of dimethoxyphenol	Optimum pH of 9 for the substrate 2,6-dimethoxyphenol	[152]
*Coprinopsis cinerea*	Dye decolorization	High activity towards 2,6-dimethoxyphenol at pH 8-8.5; decolorization of 87% of indigo dye at pH 7.0–7.5	[153]
